# Facilitators and Barriers to the Sustainability of a School-Based Bullying Prevention Program

**DOI:** 10.1007/s11121-022-01368-2

**Published:** 2022-04-25

**Authors:** Sanna Herkama, Mari Kontio, Miia Sainio, Tiina Turunen, Elisa Poskiparta, Christina Salmivalli

**Affiliations:** 1 INVEST Flagship Research Center, University of Turku, Turku, Finland; 2 Department of Psychology and Speech-Language Pathology, University of Turku, Turku, Finland; 3 Education Division, City of Helsinki, Finland; 4 Faculty of Education and Psychology, University of Jyväskylä, Jyväskylä, Finland

**Keywords:** Bullying, Intervention, Implementation, Prevention, Program, Qualitative, School, Sustainability

## Abstract

The long-term sustainment of bullying prevention programs has rarely been investigated. This study addresses this gap by identifying facilitators and barriers to the systematic implementation of KiVa antibullying program in real-life conditions, after an evaluation trial. The study is based on focus group interviews with teachers from 15 Finnish primary schools implementing the KiVa program. The schools were selected based on the annual KiVa survey data, with the criteria of long-term involvement in delivering the program and reaching successful outcomes in terms of decreasing trends in bullying and victimization. By utilizing thematic analysis, we identified program-related, organizational, and contextual facilitators and barriers to sustainability. The results stress the importance of organizational factors in promoting program sustainability.

Bullying at school, defined as repeated negative actions directed at a physically or socially less powerful person (Olweus, 1993), has received plenty of public attention during the past decades and quite a few approaches to tackle the problem have emerged. Typically, research on bullying prevention has concentrated on evaluating the main effects of bullying prevention programs. A recent meta-analysis indicates that these programs reduce the prevalence of self-reported bullying perpetration on average by 19–20% and victimization by 15–16% (Gaffney et al., 2019). Importantly, empirical studies have pointed out that the program effects tend to be stronger the longer a particular program has been implemented (Huitsing et al., 2020; Olweus et al., 2019).

However, program efficacy alone does not ensure program sustainment and schools typically do not deliver all of the program components over time. For instance, the KiVa antibullying program developed in Finland has been shown to be efficacious in reducing bullying and victimization (Kärnä et al., 2011a, b) and it was successfully disseminated across the country reaching more than 90% of the Finnish basic education schools. Nonetheless, utilizing the annual student survey as a proxy for program participation, Sainio et al. (2020) found that almost half of the schools had a decreasing trend in active involvement across 7 years (i.e., they stopped replying the survey). Moreover, the results of the annual survey have revealed that there is variation between the schools and across the years in how the KiVa program is being implemented (e.g., Which program components are delivered and to which extent?, Saarento et al., 2017; Salmivalli, 2009). Thus, the sustainability of evidence-based programs is a serious concern given the effort and investment placed in the development, initial evaluation, and scaling up of such programs. It is quite evident that even the most effective interventions will fail if they are not implemented properly: if resources are not devoted to their sustainability, the initial investments are wasted and the full potential of the programs is not realized. For example, a RCT conducted in Wales found no statistically significant effect of the KiVa program on child reported victimization or secondary outcomes (Axford et al., 2020). The authors suggest that low implementation fidelity might partly explain the results. This even further stresses the importance of understanding the facilitators and barriers to program implementation.

Implementation of a program or a policy can be viewed as a process. The Exploration, Preparation, Implementation, Sustainment (EPIS) framework (Aarons et al., 2011) is based on this idea and divides implementation into four phases. A recent review concluded that the sustainment phase is considerably less studied compared to the implementation phase (Moullin et al., 2019). This is also the case in the bullying prevention field: To our knowledge, only few studies (Leadbeater et al., 2015; Sainio et al., 2020) have looked specifically at the sustainability of practices to prevent peer victimization and bullying.

Overall, sustainability refers to continued use or delivery of program components and activities (Moore et al., 2017; Scheirer & Dearing, 2011; Shoesmith et al., 2021). Also, concepts of continuation, confirmation, maintenance, durability, institutionalization, incorporation, integration, and routinization have been used to describe the multifaceted phenomenon of sustainability (Savaya & Spiro, 2012; Scheirer & Dearing, 2011). As these concepts imply, sustainability indeed should not be understood as an end-point but rather as a process. In the context of intervention studies, one critical time point for sustainability in this process is the moment when the evaluation period is over and the schools are supposed to sustain the intervention practices under real-life conditions, that is, in the absence of the intervention study and program developers' support. Furthermore, the importance of program fidelity, i.e., implementing a program as designed by the program developers (Dane & Schneider, 1998), has been emphasized based on the assumption that not following the given guidelines may compromise the outcomes achieved. But, in order to sustain a certain practice, it needs to “fit in” with the existing structures. Thus, the implemented program may evolve or adapt while still continuing to produce the anticipated benefits (Moore et al., 2017), and the adaptation of the intervention to better fit the context is important for the sustainment of it (Herlitz et al., 2020).

Previous studies on health interventions in general (e.g., Scheirer, 2005; Scheirer & Dearing, 2011; Shediac-Rizkallah & Bone, 1998) and school-based programs promoting socio-emotional skills and mental health more specifically (e.g., Andreou et al., 2015; Leadbeater et al., 2015; Woodbridge et al., 2014) have identified factors potentially influencing program sustainability. They may originate from (1) the project or program itself, (2) organizational setting, and (3) the broader context including the community, as well as the socioeconomic and political landscape (see Han & Weiss, 2005; Scheirer, 2005; Shediac-Rizkallah & Bone, 1998).

Considering *program-related factors*, the external implementation support, for instance from the program developers, as well as program characteristics such as its effectiveness, feasibility, and flexibility, have been suggested to be important factors in promoting sustainability (Andreou et al., 2015; Leadbeater et al., 2015; Sanford DeRousie & Bierman, 2012; Woodbridge et al., 2014). Although initial training has not been a straightforward predictor of practice maintenance (Haataja et al., 2015), the staff, quite naturally, needs to have sufficient knowledge about the program and to believe that the program works, in order to start in the first place (Han & Weiss, 2005; Woodbridge et al., 2014). Moreover, the basic structure of the program and the implementation of it should not create extensive burden to the staff and should be flexible enough to be fitted in the local school culture (Andreou et al., 2015; Han & Weiss, 2005; Scheirer, 2005).

The *organizational setting* can influence the capacity of the school to adopt and sustain prevention programs. Even purely demographic factors, such as school size, may influence program sustainability in various ways. In some studies, larger schools have been found to be more likely to sustain programs possibly simply because they have more resources (McIntosh et al., 2016a, b; Sainio et al., 2020). Interestingly, teacher reports from large public schools in low-income attendance zones indicated that large schools specifically struggled with resources and were vulnerable to staff turnover (Sullivan et al., 2021), which could potentially be a barrier to sustainability due to reduced commitment (Andreou et al., 2015). The fit between values of the program and the priorities of the school influences the allocation of time and other resources to implementation (Andreou et al., 2015; Leadbeater et al., 2015; Sanford DeRousie & Bierman, 2012; Scheirer, 2005). Also, resources in general, such as staff, money, and having enough time to train the staff and implement the program during hectic school days, have often been mentioned in previous studies as important factors influencing implementation (Bruening et al., 2018; Limber et al., 2004; Sullivan et al., 2021). It seems that handling acute cases of bullying calls upon fairly quick responses and thus time allocation is crucial (Cunningham et al., 2016a, b).

Program fidelity can influence the attained outcomes and benefits in bullying prevention (Cross et al., 2019; Haataja et al., 2014; Swift et al., 2017), thus contributing to a positive cycle where, for example, the realization of reduced prevalence of bullying may encourage further implementation. Moreover, the delivery of various program components can significantly promote the creation of a unified school culture and code of conduct, which in turn may facilitate sustainability (Leadbeater et al., 2015). Importantly, Sainio et al. study (2020) indicated that a “flying start,” that is, a high level of implementation during the first years predicted program sustainment over time. At best, putting effort in implementation at an early stage can support sustainability in the long run. In addition, positive connections between key stakeholders (e.g., students, teachers, and parents; Coyle, 2008; Limber et al., 2004) and relationships among staff members may facilitate program sustainment.

Implementation requires coordination at the school level (Leadbeater et al., 2015; Sainio et al., 2020) and also headmaster and administrative support are necessary (e.g., Ahtola et al., 2013; Cunningham et al., 2016a, b; Haataja et al., 2015; Sanford DeRousie & Bierman, 2012; Sullivan et al., 2021; Woodbridge et al., 2014). In addition, staff buy-in and ownership are essential for long-term maintenance of the program (Andreou et al., 2015; Leadbeater et al., 2015; Sainio et al., 2020); a program needs motivated staff members who advocate it within the school. Overall, engaging the whole school appears important (Leadbeater et al., 2015; Sainio et al., 2020). That is, raising awareness and informing students, staff, and parents about the program promote program longevity. Finally, the use of data as feedback for the schools to clarify decision-making on program continuation has been mentioned as a potential facilitator of sustainability (Andreou et al., 2015; Scheirer, 2005). Also observing and experiencing the benefits of the program, such as problem behavior decreasing in everyday life at school, can contribute to program sustainability (Sanford DeRousie & Bierman, 2012; Woodbridge et al., 2014).

In regard to broader *contextual factors*, the overall socio-economic and political landscape shapes school policies and priorities and thus can directly or indirectly influence the allocation of resources to specific programs (Han & Weiss, 2005; Shediac-Rizkallah & Bone, 1998). Both direct provincial support as well as enhanced flexibility of the national-level core curriculum have been found to enhance the implementation of a school-based mental health program (Leadbeater et al., 2015). Budget cuts, competing policy initiatives, and new programs, in turn, have been viewed as barriers to sustainability (Leadbeater et al., 2015; Woodbridge et al., 2014).

The factors reviewed above interact during the implementation process (Scheirer, 2005). As Han and Weiss (2005) point out, some of them can be more relevant at pre-implementation phase and determine whether the program is adopted in the first place. For instance, following teachers implementing the KiVa lessons during one school year, Haataja et al. (2015) showed that believing in program effectiveness predicted higher implementation in the very beginning. However, it was the headmaster support that eventually differentiated the teachers who continued delivering lessons from those who started high but then surrendered. In the end, the accumulation of the facilitators and barriers to sustainability likely determines whether the program is maintained in the school’s everyday practices (Han & Weiss, 2005). Moreover, the studies reviewed above, recent reviews on sustainability in the context of school-health interventions (Herlitz et al., 2020; Moullin et al., 2019; Shoesmith et al., 2021), and studies on program implementation in general — not on sustainability itself — in the bullying prevention field (see, for example, Coyle, 2008; Cunningham et al., 2016a, b; Limber et al., 2004; Sainio et al., 2020; Sullivan et al., 2021) suggest that several organizational factors are essential to program sustainment. Thus, we expect organizational factors to be strongly featured also in our study, especially because we are investigating sustainability from the teachers’ point of view.

To our knowledge, factors influencing sustainability of bullying prevention programs specifically are yet to be explored. In this study, we address this gap by identifying facilitators and barriers to sustaining KiVa antibullying program in Finnish primary schools during 2009–2017. The study builds on Sainio et al. (2020) quantitative study, where a group of schools persistently implementing the KiVa antibullying program was identified. This study deepens the understanding of what it takes to sustain a complex antibullying program under real-life conditions by utilizing qualitative focus group interviews with teachers representing such persistent schools. We pay attention to program-related, organizational, and contextual facilitators and barriers to sustainability.

## Method

### KiVa Antibullying Program

KiVa antibullying program aims to reduce bullying in basic education (grades 1–9 in Finland, ages 7–15). The program was developed at the University of Turku, Finland, during 2006–2009 (more on the program development in Herkama et al., 2017). The program development and the large nationwide dissemination were funded by the Finnish Ministry of Education. Several evaluation studies conducted so far in Finland (e.g., Kärnä et al., 2011a, b) and in other countries (e.g., Huitsing et al., 2020; Nocentini & Menesini, 2016) indicate the program to be efficacious in reducing rates of being bullied and bullying others. After the evaluation period, KiVa was disseminated across Finland from 2009 onwards. The program reach was close to 100% and the uptake at best over 90%. The data from the first year of nationwide implementation (Kärnä et al., 2011a) and from the KiVa annual survey (Salmivalli, 2009) indicate that self-reported bullying and victimization have decreased across years in KiVa schools (see also, Sainio et al., 2019). Also, the National School Health Promotion Study indicates that the prevalence of bullying is decreasing at the national level from 2010 onwards (Finnish Institute of Health and Welfare, 2019), although there might be other contributors to this development besides KiVa.

KiVa is a multi-component program based on the participant role approach (Salmivalli et al., 1996). It includes both universal actions targeted to all students and indicated actions to be utilized when acute cases of bullying emerge. The universal actions include (1) student lessons and online games targeted for specific year groups (1, 4, and 7), (2) visible KiVa symbols (posters and recess supervisor’s vests), (3) ready-made materials for events (Kick-Off for students, Back-to-School Night for parents, information to be delivered in Staff-meetings), and (4) teacher manuals, parent's guide, presentation graphics, and newsletters introducing the program. The indicated actions involve clear guidelines to tackle acute cases of bullying. Each school implementing the program should have a KiVa team (consisting of 2–4 staff members) responsible for addressing all bullying cases. Also, the schools are advised to participate in the annual student and staff online surveys which provide information regarding the level of the program implementation (reported by teachers) and the outcomes achieved (e.g., the experiences of being bullied and bullying others, reported by students).

### Selection of Schools

Our criteria for contacting schools were the following: (1) sustained program implementation over the years, (2) ongoing participation at the time of the recruitment, and (3) overall success in reducing the prevalence of victimization and bullying across time. Moreover, we decided to focus on primary schools (e.g., grades 1–6, age group 7–12). Furthermore, we restricted the sample to schools identified as *persistent* in an earlier study based on student responses to annual KiVa surveys (*n* = 617, Sainio et al., 2020). These schools had started KiVa already in 2009 or in 2010, and thus could provide long-term perspective on implementation. In order to ensure that the schools still actively implemented the program, we focused on schools in which the staff had responded the annual KiVa surveys the previous spring (i.e., responses in 2016 surveys from a KiVa team member as well as at least half of 1st and 4th grade teachers that were expected to respond). Also, the schools selected for the study were required to be registered program users during the school year 2016–2017 (i.e., the first year the annual license fee was introduced which reduced the number of KiVa schools in Finland). From this restricted sample of 86 schools, we identified schools with successful reduction of bullying/victimization problems, indicated by a descending slope of the combined victimization and bullying variable steeper than the average slope among all schools replying to the student questionnaire 2009–2016. Approximately half of the 86 schools met this criterion. We then contacted altogether 22 schools, one at a time, until 15 schools willing to participate in the study were found (e.g., seven schools contacted did not participate). The schools were contacted in an order that would maximize the diversity of the sample (countryside vs. urban schools, school size). The schools in the final sample were located in different regions of Finland and represented both urban and suburban areas. The average school size was 277 students (ranging from 83 to 492).

### Focus Group Procedures

Altogether 15 focus group interviews were conducted in spring 2017 by the first three authors (all certified KiVa trainers and familiar with the program). The focus groups included (1) a staff member, who had been actively involved with the implementation of KiVa from the very beginning on, (2) former and/or current active KiVa team member, and (3) a staff member currently responsible for the program coordination in the school. Depending on the school, 2–5 staff members participated in the interview during regular school hours. Most of them were classroom teachers, but also special education teachers and one school social worker participated. Each participant was offered a free KiVa teacher manual, and the school staff was also given reduced admission to upcoming national KiVa training days organized by the program developers. The permission for the focus group interviews was granted by the schools’ headmaster, or in few cases by the municipal board, and all participants provided an informed consent.

During the focus group interviews, the participants were asked to describe freely the implementation of KiVa across years. Clarifying questions were presented on the way (e.g., specific questions on delivering various program components, trainings participated in). Also, several questions were posed on sustainability specifically (e.g., What promotes program delivery in everyday life at school? What hinders it? How to ensure that KiVa becomes a part of the school’s everyday practices? How to ensure that KiVa is sustained over several years in your school? What motivates staff members to deliver the program in the long run?). The interviews were kept as informal and conversational as possible. The interviews were recorded and transcribed verbatim. Their length varied from 1 h 1 min (roughly 35 pages transcribed text, 12pt, 1.5 spacing) to 1 h 36 min (roughly 60 pages transcribed text, 12pt, 1.5 spacing).

### Analysis

The data were analyzed using thematic analysis (Braun & Clarke, 2006), and it was conducted with the aid of ATLAS.ti 7.5.1, a qualitative data analysis software. The data analysis consisted of four phases which were (1) preliminary views on data, (2) creation of a coding frame, (3) coding of the research material, and (4) creation of shared understanding. The first three authors (senior researchers) were responsible for the analysis. First, the transcribed focus group interviews were transferred to ATLAS.ti software and the first two authors, experienced qualitative researchers, went through the data independently to get acquainted with it. Second, they met with the third author to discuss and trained her for the task, and the three of them collaboratively created a coding frame. A theory driven standpoint was chosen and the (1) program-related, (2) organizational, and (3) contextual facilitators and barriers to sustainability created the core of the coding frame. Further informed by preliminary reading of the research material, previous literature, and practical knowledge on KiVa, various sub-themes and also sub-sub-themes of facilitators and barriers were identified and added to the coding frame. Through series of discussion, the essence of each theme was identified and conflicting views were resolved.

Third, the data were coded independently by the first and second author utilizing the created coding frame. In practice, all relevant text segments were identified and connected to specific themes. The length of the identified text segments varied between a sentence and several sentences. The text mass was fairly large including descriptions connected also to program implementation on daily basis and, therefore, segments referring to sustainability specifically were paid special attention to. Fourth, all the text segments identified by the researchers were compared in order to clearly identify the key findings. Again, the core of each identified theme was pondered upon and the boundaries as well as the essence of each theme were clarified. The program-related, organizational, and contextual facilitators and barriers to the sustainability of a bullying prevention program are presented in Table 1.

**Table 1:** Facilitators and barriers to sustainability identified in the theory-driven thematic analysis

Theme	Sub-theme	Sub-sub-theme
Program-related	Usability facilitating program delivery	Systematic program structure
		User-friendliness
		Adaptability and program fidelity
	Instructional capacity	N/A
	Supportive role of program developers	N/A
	Realistic expectations and recognition of program boundaries	N/A
Organizational	Motivation to continue program delivery	Values
		Commitment
		Benefits in everyday life
	Administration and organizational development enhancing long-term sustainment	School level coordination
		Lack of resources
		Headmaster’s support
		Human resource management
		Importance of training
	Organizational culture supporting sustainability	Symbols
		Supportive school climate
		Integration to everyday practices
Contextual	National core curriculum as framework for bullying prevention across time	N/A
	School’s bullying prevention plan guiding actions taken in practice	N/A
	Positive and negative media attention	N/A

## Results

### Program-Related Facilitators and Barriers to Sustainability

During the interviews, teachers referred to (1) usability, (2) instructional capacity, (3) support from program developers, and (4) realistic expectations and recognition of program boundaries as program-related factors affecting program sustainability.

#### Usability Facilitating Program Delivery

##### Systematic Program Structure

According to the teachers, systematic program structure facilitated program delivery in various ways. It offered a solid background for antibullying work, promoted program delivery in everyday life, “strengthened” the actions taken, and lessened the tendency of individual teachers to “act haphazardly.” In addition, one teacher pointed out that clear guidelines on how to address acute cases of bullying can lower the threshold for intervening and increase teacher’s confidence to intervene. Furthermore, another teacher explained that also the allocation of resources, such as time for anti-bullying work, increased in their school along with the adoption of a systematic approach. This teacher specifically stressed the importance of clear program structure when tutoring new staff members:

… And then we have clear steps in our school for how to intervene. It makes it really easy. We are able to tell that this is the intervention model we use, to a newcomer, for example, and they can familiarize themselves with it. (Teacher 1, School 13)

##### User-friendliness

User-friendliness was considered as a necessity for program delivery — “the easier the program is to use the more it will be implemented” stated one of the participants. In this discussion, the participating teachers declared fast and in unison what in their opinion promotes program implementation:

Teacher 1: Good materials.

Teacher 2: Good materials.

Teacher 3: Materials being updated.

Teacher 2: Yes, materials being updated.

Teacher 1: Materials being easy to get the hang of.

Teacher 2: Materials being available.

Teacher 1: In my opinion, the good thing about KiVa is that the materials are not only available in paper but also online. (School 7)

Thus, teachers valued materials which were user-friendly. It is important to notice that the schools participating in this study were preselected among the ones that had managed to keep up with the program delivery over time. However, barriers for sustainability were also recognized and reflected upon during the interviews. For instance, utilizing electronic services were regarded as difficult by some respondents (e.g., because of technical problems, and lack of laptops or tablets in the school) and this was seen to risk the continued use of them.

##### Adaptability and Program Fidelity

The participants noted that a bullying prevention program “needs to be flexible” in order to be delivered and one teacher pondered that fidelity is expected but supposedly “some adaptations are allowed.” Furthermore, the rich and versatile lesson material was described to enable the setting of various educational goals depending on the needs of the students, the teaching styles of the teacher, and the framework set by current curriculum. Nevertheless, although the importance of program adaptability was raised up, the teachers also made references to program fidelity. For instance, one teacher mentioned that starting by following the instructions precisely was one of the key factors in adopting the program and eventually finding the ways to “fit the program to the everyday life at school.” In another school, the teachers described KiVa to be the most solid program they had been following in the school for several years. Thus, the importance of both, program adaptability and program fidelity, were mentioned in connection to sustainability.

#### Instructional Capacity

At best, interventions can carry significant instructional capacity which in this case refers to the educational potential of a bullying prevention program. As such, an intervention can have the capacity to inform the practitioners about bullying as a phenomenon and offer practical tools for prevention, facilitating sustainability. This came up in some cases: Understanding the program foundations and implementing the program in practice led to some profound realization. First, few participants pondered upon how the program implementation might have influenced the understanding of the bullying phenomenon in general (e.g., bullying as a group phenomenon, something that should be taken seriously, and not a problem or failure of one single teacher) as this quote illustrates:

But maybe, the KiVa program also opened the teachers’ eyes, brought some courage to intervene. So that it is not only about the bully and the victim, but also about looking more broadly at the role of everyone else [in the classroom]. (Teacher 2, School 1)

Second, some participants described that through KiVa, they had understood more in-depth the importance of preventive work: “Then it is not only about fighting the fire when something happens but preventive work instead.” Third key learning for some was that addressing acute bullying cases is not about punishment or accusations, but rather about helping children to improve their social skills and enabling behavior change. Thus, it appeared that implementing a bullying prevention program also served as a way for educators to learn more.

#### Supportive Role of Program Developers

A few participants explicitly pointed out that they valued the program being evidence-based, nationally implemented, and being investigated scientifically instead of being a set of procedures invented by a single teacher. Furthermore, the teachers acknowledged the support from and contact with the program developers to be important. Specifically, they longed for new materials, trainings, meetings, and specific campaigns organized by program developers. Such events were experienced as refreshing and described as motivating. Also being connected with the program developers was seen as a motivating factor: “ … The motivation is higher if there is the connection to the program developers, the ones that have designed the program. If the connection is strong, I think that is the key.”

#### Realistic Expectations and Recognition of Program Boundaries

During the interviews, teachers shared stories about challenging bullying cases they had faced, thus expressing the limitations of the program in erasing all bullying. The challenging cases typically included several parties, were long lasting, and took plenty of resources. In many cases, it was evident that the students involved needed professional help other than the school alone could provide. Also, family-related problems were mentioned (e.g., parents having been bullied themselves, serious problems in the family, parents’ attitudes or views on bullying that were unhelpful). Co-operation with various stakeholders, such as parents and multiprofessional working groups, was seen to be important when dealing with such cases.

Thus, it was clear that KiVa program did not solve all bullying cases, and although the program’s good reputation was mentioned as an important facilitator of delivery during the first years, this reputation was brought up as a double edged sword when failing to solve challenging bullying cases. Specifically, teachers pointed out to the unrealistic expectations towards the program among school personnel, parents, or in some cases raised in public (e.g., social media) discussions. Most often the teachers referred to parents expecting the program to remove all bullying from the school. As one teacher quite aptly stated, “KiVa is not about miracles but rather about common sense.” Such realization may be crucial in order to maintain the practices despite facing problems.

### Organizational Facilitators and Barriers to Sustainability

The organizational features identified were connected to the following broad sub-themes: (1) Motivation to continue program delivery, (2) Administration and organizational development enhancing long-term sustainment, and (3) Organizational culture supporting sustainability.

#### Motivation to Continue Program Delivery

##### Values Promoting Sustainability

Some teachers emphasized that bullying prevention was inherently meaningful for them; bullying prevention was said to be “a calling” and a few teachers described “being in their vocation.” This teacher described how strongly she felt about the program from the very beginning on: “It somehow came in the training, we were so excited, we got to do some acting and. So we had a passion for it, so I have to say it was close to my heart to implement it and still is.” Furthermore, participants stressed the importance of shared values. The importance of children’s and community’s well-being was emphasized in almost every focus group interview, and the interviewees considered student well-being, safe school environment, and/or bullying prevention to be essential part of the school values. As one teacher put it: “Bullying prevention is a priority in our school.” Overall, understanding how important the well-being of students is and how being bullied jeopardizes their healthy development were seen essential elements in facilitating commitment and motivation to continue program implementation. In some interviews, the teachers, nevertheless, pointed out that not all teachers share the same values and this should be accepted. Also, resistance to change was mentioned during the interviews: New things are not always well-received.

##### Importance of Commitment

Indeed, commitment was mentioned often in the interviews as being an essential part of sustainability: If the teachers are not committed, bullying can take longer to solve, delivery of program components may be at risk, and the program does not become an integral part of the everyday life at school. One interviewee explained how the behavior of a teacher who is not fully committed can decrease the quality of program delivery: “And especially if the teacher is not committed, delivers the lessons begrudgingly, and does not stand behind the ideas, well then, the children will notice that this is not such an important thing.” Importantly, the teachers stressed that it is not enough that a few staff members, such as the KiVa team members, are committed. The program needs to be adopted by the majority of teachers in order to be sustained in the long run. Finally, financial compensation in terms of the employer paying for one extra hour per month or giving exemption from common duties such as recess monitoring was often mentioned in connection to commitment. Teachers regarded compensation as a signal of support and recognition from the school management that antibullying work is valued.

##### Recognizing the Benefits in Everyday Life

The results emphasize how important and motivating it is for teachers to notice the benefits of program delivery in their daily work. According to the teachers, program implementation had the potential to influence everyday life at school in many positive ways. Bullying prevention was seen to be connected to increased well-being of students, better functioning classrooms, feelings of relief after a successful intervention, increased trust, and being rewarded after successful intervening. One teacher explained that it can be seen on the faces and appearance of the students that they feel safe at school. For her, this was the best sign of success in bullying prevention. Furthermore, positive student feedback and enthusiastic participation and profound discussions during KiVa lessons were described to promote further lesson delivery. In addition, noticing that the skills practiced during the student lessons transferred to real life, for example, when a student intervened in bullying situation, was considered motivating. The rewards in the form of successful outcomes were seen as reasons to continue with the program and they were at times described to be very emotional moments as this teacher describes:

Of course those moments can be very impactful when you see the situation unravel and, in the best case, the children begin to recover when the bullying stops. [It happens] both in the victimized child and in the one who had been doing the bullying, and those moments can be very emotional. At times I have also had tears in my eyes. (Teacher 1, School 7)

Also the results provided by the annual KiVa survey were seen to facilitate program delivery. In some cases, the results were seen to build up within time: over several years of persistent preventive work, the efforts taken started to carry out positive results in the form of decreased level of being bullied and bullying others. As one teacher stated, the results “tell something about the effectiveness of the program, working culture of the school, and about our own success.” The survey results were utilized in evaluating schools bullying prevention work and they were seen to offer valuable information on actions taken. In one school, for instance, the survey results yielded an increase in the student reported bullying prevalence along with the decreased level of program implementation. This information led to improved delivery of the program and “getting our act together,” as described by teachers. In some schools, the program implementation was evaluated yearly, for instance, by selected KiVa team members, school’s student welfare team, or local education and culture committee. Nevertheless, the survey results were considered as an underused resource — the results could be utilized more when planning and evaluating antibullying work.

#### Administration and Organizational Development Enhancing Long-term Sustainability

##### School Level Coordination

Teachers described the school level coordination being a necessity for sustained implementation of a complex prevention program. In some schools, coordination was more frequent and involved several staff members, whereas in others, the responsibility laid on the shoulders of one person, sometimes for many years. In any case, the teachers emphasized the role of active staff members. They often kept encouraging others, invited colleagues to participate, and coordinated program implementation. One teacher even described the way of informing others about the KiVa surveys being available for students in a very strict and straightforward way: “There is x amount of time left, book the time slot in this calendar, *do this* [italics added].” Some teachers stated that if the staff members do not share the same view on how to deliver various program components in practice, the motivation for implementation may decrease. Taken together, from the sustainability point of view, it is important to plan well how to deliver the program and also communicate this to all staff members.

##### Lack of Resources

One of the biggest obstacles for sustained program delivery seemed to be lack of resources — especially time. Typically, teachers stated that they struggled to find time to prepare and deliver the preventive student lessons and to handle the acute cases of bullying: “And actually, of course there are exceptions and special cases that need to be agreed on, they are, like, like we have such a little time here at school, so then the lack of time becomes the problem.” But also solutions were described. For instance, scheduling monthly preventive lessons at the school level, choosing KiVa team members wisely (e.g., teachers with more flexible schedules, such as special education teachers), utilizing the help of assistant and resource teachers, and having a fixed time slot to handle acute cases of bullying were described to be well-functioning practices for promoting sustainability. Along with time and personnel resources, teachers mentioned recently introduced program-related annual costs as a potential risk for program continuation. They were pondering upon what would happen if the funding was cut down. In general, allocating enough resources — time, personnel resources, and financial support — was seen vital and failure to provide the teachers with these resources seemed to form a considerable risk for sustainability.

##### Headmaster’s Support

Support from the headmaster was described to be a cornerstone of program sustainability. The role of the headmaster was seen crucial in resource allocation and in offering important backup for the KiVa team with challenging bullying cases like this respondent describes:

What really enables it [implementation] is the headmaster’s support. The fact that the headmaster says it is OK to have a substitute teacher when you have to take care of an acute bullying case and also the feeling of having a back-up is also important. (Teacher 3, School 10)

Also, the headmasters were described as the ones informing and reminding the staff members of the importance of bullying prevention (i.e., setting priorities) and giving appreciation for the work accomplished. Interestingly, teachers also pointed out that direct orders are at times necessary, otherwise nothing happens: “So, if we are not told to do something, well, then we don’t do anything.”

##### Human Resource Management

Human resource management was brought up as a prerequisite of sustainability: New staff members need to be aware and part of the bullying prevention practices and key staff members need to be rotated every now and then in order to make sure the program does not get too personified or too heavy to carry on. Rotating teachers in key positions was also seen to increase commitment, involvement, and learning over time. In some schools, the program delivery had suffered greatly due to staff turnover, especially if the key persons had left the school with a short notice. Thus, according to the teachers, there needs to be a right balance between competent and experienced teachers and newcomers in key positions. These two participants reflect on the current situation with staff turnover and orientation for the new staff members:

Teacher 2: But, this changing of staff has become to the picture during the past years…

Teacher 1: [interrupts]: Yes, it messes things up.

Teacher 2: It messes things up, like we have had no time to brief newcomers or so. (School 6)

##### Importance of Training

Need for training was highlighted in the interviews. One teacher declared that the trainings had brought “more flesh on the bones,” and another participant explicitly stated that training may increase commitment: “Training for as many as possible. It is easier to commit to a program if you understand the basic principles and the philosophy behind it. Just briefly hearing about it in a teacher meeting might not lead to commitment.” The teachers also stated that participating in training does not change anything if actions are not taken in practice. Understanding the basis of the program thoroughly and learning to deliver the lessons and organize the KiVa team discussion were described to take some time. Furthermore, the teachers emphasized that a single teacher participating in a training, although very committed and enthusiastic, cannot change the whole school community. Thus, the need for several teachers to be trained was highlighted, since it facilitates discussion, commitment, and planning of future activities.

#### Organizational Culture Supporting Sustainability

##### Symbols

Teachers considered various symbols being essential in creating a unified antibullying culture in the school. Simple acts such as wearing recess supervisor’s vests, having posters on the walls, singing KiVa songs (e.g., a specific song chosen to symbolize KiVa and to be sang in whole school events), and various get-together events organized by the KiVa team were considered important in strengthening the antibullying culture. Talking about KiVa and reminding students that we are a KiVa school where bullying is not tolerated seems to have a crucial role in raising awareness among students. According to the teachers, this should be done regularly in order to strengthen the key messages. This teacher describes how also children notice these symbols:

One concrete example, how it [KiVa program] has become a part of life in our school are the research supervisor vests which are a visible sign of KiVa also for children. So, when there was a substitute [teacher] out there without a vest, a first grader reported that maybe they did not tell that man that in our school he is supposed to wear the vest. (Teacher 3, School 10)

##### Supportive School Climate

A generally warm and genuine school climate was perceived as promoting bullying prevention, and well-functioning work community was seen as a great source of support in the long run. One participant evaluated their own work community to be a place where everyone can share their problems and find solutions together. Another teacher described her work community by saying “there is no need to pretend anything” and “in our teachers’ lounge you can ask everything.” Thus, for teachers, it seemed to be important to be able to rely on colleagues and get support when facing problems. Teachers also mentioned some best practices that supported program delivery (e.g., delivering KiVa lessons together with another teacher or doing KiVa team discussions together with a close colleague); these examples highlight the importance of collegial support.

##### Integration to Everyday Practices

The participants made references to the existing school structures and practices. One teacher, for instance, noted that a bullying prevention program “has to fit in the school” in order to be sustained, and another one emphasized that it was easy to start with KiVa because the program aligned with the already existing values of the school. Thus, a program needs to somehow fit to the current school structures in order to be adopted in the first place. In the long run, the integration of the program to the everyday life at school seems to become an essential for program longevity. Indeed, the participating teachers made references to KiVa becoming established, or “a natural part of the school culture.” Interestingly, teachers were also pondering upon what happens when the program is being routinized: In one interview, the teachers discussed whether the program should be more visible since it had become a part of everyday practices but was not that often referred to in different occasions. The teachers wondered to which extent the students recognized KiVa and realized being in a school where bullying is not tolerated. In another school, KiVa lessons were integrated into different subjects but were no longer called KiVa lessons. Consequently, the program was part of the everyday practices, but the adaptations made it also somehow invisible: KiVa was not that often talked about or present in happenings and in everyday talk in classrooms. These examples reflect the need for raising awareness and promoting antibullying values over time.

### Contextual Facilitators and Barriers to Sustainability

During the interviews, also contextual factors facilitating program sustainability across years were referred to. More precisely, teachers described (1) national core curriculum, (2) school’s bullying prevention plan, and (3) media attention, as facilitating sustainability but also setting barriers to it at times.

#### National Core Curriculum as a Framework for Bullying Prevention Across Time

The nationwide dissemination of KiVa started in 2009. As one teacher stated, there was “room for a program like KiVa” in schools at the time. There was a need for a program offering tools for bullying prevention and socioemotional learning during those years. Since the program had become very well-known publicly, teachers did not want to miss their chance to get familiar with it, and many reported that they had taken part in the initial training days which were offered free of charge (e.g., 2–3 teachers/school). The national core curriculum for basic education (released in 2014, and put in action in 2016 in grades 1–6; see Finnish National Board of Education, 2016) even further emphasized the importance of social skills. This was seen to be in line with the basic foundations of KiVa, and facilitating its adoption and sustainment, as this teacher describes: “The new curriculum of course, it encourages towards something like this. … Somehow it [KiVa] fits to the spirit of the new core curriculum.” In addition, teachers described that the importance of student well-being had been given more emphasis during the past years and this was also considered to support program delivery.

Currently, there seems to be more competition between different programs and projects in basic education. As teachers pointed out, the context in which interventions are to be implemented has changed remarkably compared to the first years of nationwide dissemination of KiVa. One teacher explained that on top of regular teaching, there are currently many projects the school is involved with, but time is limited. The interviewees explained that these types of projects drain the staff, and at times, they create negative attitudes towards new responsibilities and programs. Since there are plenty of other competing initiatives, setting bullying prevention as priority and devoting resources to it was seen even more important now than before.

#### School’s Bullying Prevention Plan Guiding the Actions Taken in Practice

During a few interviews, teachers mentioned that one reason to continue with the program was the fact that KiVa was stated to be part of school’s official bullying prevention plan, which gave a signal to individual teachers that program implementation is important. When asked to reflect on how to facilitate sustainability of a bullying prevention program, one teacher stated that “We have, for example the action plan. We state each autumn that KiVa is part of this plan.” Indeed, the legislative amendments made in 2013 made it compulsory for each institution offering basic education in Finland to have a plan for bullying prevention and intervention (Pupil and Student Welfare Act (Fin.), 2013). In some cases, KiVa program had been officially named as the primary method even in the municipality’s bullying prevention plan. This offered a solid base for the implementation of KiVa, facilitating program sustainability.

#### Positive and Negative Media Attention

The last contextual factor described by teachers was media attention. Over the years, the KiVa antibullying program had received a lot of media attention in Finland — both positive and negative. More recently, the program had been discussed publicly when severe bullying cases were dealt with in the social media or in the news. Even though the teachers, in general, told that they try not to let the bad news or criticism affect them too much, some of them reported being frustrated at times about people’s way of generalizing schools’ (in)activity in preventing and tackling bullying, for instance, in social media. Due to professional confidentiality, they felt it was not possible to equally engage in discussion and respond to, for instance, accusations as the following discussion on social media influence points out:

Teacher 4: … Or then there are parents attacking you sharply that nothing has been done although their child has been bullied for years and in those cases teachers’ hands are totally tied.

Teacher 1: No chance to defend oneself.

Teacher 4: Or like, we cannot defend ourselves, no, we cannot tell anything, we are bound by confidentiality. (School 8)

In such cases teachers often felt that the criticism was unjustified and wrong, considering all their efforts to promote the well-being of students at school. 

## Discussion

Corroborating prior research (Han & Weiss, 2005; Scheirer, 2005; Shediac-Rizkallah & Bone, 1998), we identified program-related, organizational, and contextual facilitators and barriers to sustainability of the KiVa antibullying program. Most of the factors influencing sustainability could not clearly be determined as facilitators or barriers, but rather, they could function in both ways — both fueling and hindering program sustainment. As we anticipated, teachers referred to organizational factors (e.g., commitment, administration, school culture) more compared to program-related (e.g., program characteristics and developers) or contextual factors (e.g., national core curriculum, bullying prevention plans, media attention).

To our knowledge, this is the first qualitative study investigating the barriers and facilitators to sustaining a bullying prevention program over a longer period of time. Some findings were similar to previous qualitative investigations on earlier phases of the implementation of bullying prevention programs (Coyle, 2008; Limber et al., 2004; Sullivan et al., 2021), but the results also extend current knowledge by introducing some unique factors that need to be taken into account in order to reach long-term program sustainability.

In regard to program-related factors, three aspects are noteworthy. First, there is a need to find the right balance between fidelity (e.g., following the program guidelines) and adaptations (e.g., modifying the program to fit the existing structures). The importance of program fidelity has been stressed in previous studies (Haataja et al., 2014; Swift et al., 2017). Moreover, a recent study by Johander et al. (2021) demonstrates that modifications should be done with caution. They found that KiVa team discussions (i.e., handling acute cases of bullying) were evaluated both by teachers and students as less effective when staff members were using their own adaptations or could not specify the method they had used, compared to using the program-recommended methods. However, integrating a complex bullying prevention program to educational environment of each school often needs some adaptations. Thus, there is a clear call for research examining the elements of bullying prevention programs that can be modified while retaining their effectiveness, and these insights should be communicated to teachers.

Second, participating teachers explained how learning to use KiVa had taught them also about bullying as a phenomenon and antibullying work in general (i.e., instructional capacity). Such theoretical and research-based understanding on the phenomenon could increase teachers’ motivation to use and maintain evidence-based prevention and intervention practices. Third, developing understanding and acceptance of the program being a tool, not a solution for all problems, seems to be important for sustaining a bullying prevention program (see also Coyle, 2008) — realistic expectations are important. Otherwise, disappointment or feelings of failure can emerge among staff or other stakeholders when bullying happens regardless of preventive actions or when a challenging bullying case cannot be solved. Emerging cases, even difficult ones, are not necessarily counterevidence against program effectiveness. While more effective tools are currently being explored in the field to address the most challenging bullying cases, adequate implementation of the existing evidence-based practices and patience is needed, as change (i.e., effectively reducing bullying) might take time.

Our results demonstrating the importance of organizational factors corroborate with previous studies in the bullying prevention field highlighting the need for commitment, coordination, adequate resources, support, training, human resource management, and supportive school climate in bullying prevention (Ahtola et al., 2013; Bruening et al., 2018; Cunningham, 2016a, b; Limber et al., 2004; Sainio et al., 2020; Sullivan et al., 2021). The unique finding related to the sustainability phase of implementation seems to be the need to balance between successfully integrating the program to the school’s existing structures, while still keeping it alive and vivid across time. A particular program or policy should become a part of the school’s everyday life instead of being something “on top of everything.” However, when a bullying prevention program becomes routinized and a solid part of everyday practices, there is a risk that over time it becomes “business as usual,” taken for granted. As a possible consequence, the program disappears from the teachers’ discourse and, although partially implemented, it is easily forgotten in the long run: New teachers are not explicitly guided to program practices, resources are not allocated to program implementation, and students are not aware of existing antibullying practices if they are not specifically reminded of them.

In a broader societal picture, the legislation and policies directing schools towards high quality bullying prevention can promote sustainability. However, national level policies, legislative guidance, curriculum contents, or societal discourse alone cannot create the most important thing: a well-functioning school-level policy to prevent and intervene in bullying with enough resources that is sustained in everyday life at school over time. In addition, bullying taking place online has created new challenges for bullying prevention: Teachers find it difficult to recognize and intervene in online bullying (Bruening et al., 2018; Sullivan et al., 2021). Our study brings out a new way in which, for example, social media might influence bullying prevention and intervention: Bullying cases have been brought up in social media, but due to confidentiality, individual teachers or school representatives have to refrain from participating in the discussion and explaining what exactly has happened and which actions have been taken.

In light of the broader literature on implementation, it is important to notice that this study focuses on teacher perceptions of the sustainment of a bullying prevention program, and the results should be understood as such. We did not aim to elaborate on how implementation influences outcomes, but rather to shed light on how teachers experience the factors influencing sustaining a bullying prevention program over several years. It is more than likely that the identified factors are intertwined in complex ways: Some of them might be more influential than others, and in some cases, the presence of more than one facilitator is needed in order to sustain the program. For instance, resources do not really help if there are no committed staff members to actually utilize those resources. In general, implementation processes are contextually embedded, which may further add complexity to how these factors are apparent in one context but may not be influential in another.

The current study has used previous research and conceptualization coming from health interventions (e.g., Scheirer, 2005; Scheirer & Dearing, 2011; Shediac-Rizkallah & Bone, 1998), school-based programs promoting socio-emotional skills and mental health (e.g., Andreou et al., 2015; Leadbeater et al., 2015; Woodbridge et al., 2014), and implementation of bullying prevention programs in particular (Limber et al., 2004; Sullivan et al., 2021) as a point of reference. Taking a broader standpoint on public sector services and evidence-based practices, in addition to the process view of implementation, the EPIS framework introduces three other key components that manifest themselves in each phase of implementation (Aarons et al., 2011; Moullin et al., 2019). These components overlap with the factors explored here: The program related factors with the innovation factors (e.g., fit, developers, innovation characteristics), organizational factors with the inner context (e.g., leadership, organizational characteristics, monitoring/support, staffing, individual characteristics), and context factors with the outer context of EPIS (e.g., policies, funding, inter-organizational environments). Although the bridging factors (interconnectedness and relationships between outer and inner contexts, e.g., purveyors and intermediates) of the EPIS framework were not in the focus of this study, it is clear that the organizational and broader contextual factors are connected in the real world. Out of the themes arising from the teacher interviews, for example, social media could be seen as a factor between the societal and organizational levels.

To our knowledge, studies on bullying prevention have not utilized the EPIS framework in conceptualizing sustainment. The results of this study suggest that similar features can be found whether sustained implementation is studied in the context of a school-based bullying prevention program, or evidence-based practices in public sector services more generally. We examined the sustainability phase of implementation (see Aarons et al., 2011) in particular, but it should be noted that although being asked to elaborate on implementation from the sustainability point of view, the participants might have shared experiences of delivering the program per se without making distinctions between the phases. Furthermore, implementation process is not necessarily linear, as EPIS suggests (Aarons et al., 2011) — there might be shifts from one phase to another, and also steps backwards can be taken. Our findings suggest that this indeed is the case. What is essential over time is the school community’s capacity to adjust to changes, such as new curriculum priorities, other bullying prevention initiatives, staff turnover, various types of bullying cases, and different types of students and classrooms. Interestingly, some of the influential factors identified, such as changes in staff composition, can lead to situations where bullying prevention practices need to be re-built. Therefore, the distinction between the phases of active implementation and sustainment is not clear-cut. However, we identified many factors that are clearly important to the sustainment phase in particular, such as supportive policies in many levels (e.g., national curriculum, local bullying prevention plans), funding, staffing, and the importance of leaders’ strategic decisions and plans about how the program is delivered and supported. Interestingly, in the interviews, the teachers referred to another feature typical to the sustainment phase, namely the number of teachers committed to the program delivery and being on board (e.g., critical mass of program users). In order for a program to survive, there needs to be enough staff members standing behind it and teachers seem to be very aware of this.

### Strengths and Limitations

The strength of this study lies on its design. The schools were invited to participate based on their success in implementing KiVa program; we knew already before the focus group interviews that they had implemented the program across several years and been successful in reducing bullying. Consequently, the interviews were rich in content, and the participants were able to elaborate vividly on program implementation and produce detailed information on facilitators and barriers to sustaining bullying prevention practices, a feature often highly valued in qualitative studies. However, since the schools were pre-selected from among the ones being successful in program delivery, the results may represent the most positive experiences on implementing a bullying prevention program. Schools that have been struggling with implementation, have not followed the guidelines, or have not been able to reduce bullying may have different kinds of experiences on facilitators and barriers to program sustainment.

Another limitation is the restriction to primary schools. In Finland, lower secondary schools have a very different structure compared to primary schools, and also the KiVa lessons are organized differently in primary and lower secondary schools. Moreover, the results of this study reflect the situation in Finland, and possibly cannot be generalized across countries and school systems. Notably, the results illustrate the teacher perspective, but from the headmasters’ point of view some other factors might fuel or hinder the sustainment as suggested in previous studies in the bullying prevention field (Bruening et al., 2018; Sullivan et al., 2021). In the current study, we decided not to involve headmasters in the focus groups, in order to allow also more negative voices to be expressed, such as pondering upon headmaster’s support and resource allocation. In addition, the focus group interviews may be biased in two ways. First, as the interviewers represented the program, it is possible that the most critical voices were not heard due to social desirability. Second, recall bias is possible given the participants were asked to reflect on a 6- or 7-year time span.

### Further Studies

In this study, we examined implementation sustainability drivers and barriers in schools that had succeeded in implementing a bullying prevention program under real-life conditions up to 8 years. A clear next step would be to examine the schools failing to implement the program over time. This approach would unravel the relative importance of the facilitators and barriers now identified, and possibly reveal new ones. In addition, this approach could provide knowledge on possible factors explaining less optimal outcomes.

As previous studies suggest, some bullying cases remain unresolved (e.g., Johander et al., 2021). One possible explanation is that bullying prevention programs have potential, which is not realized under real-life conditions because their implementation falls short. In addition, plenty of resources are wasted if new intervention programs are constantly introduced instead of improving the delivery of the existing ones. Consequently, future studies on bullying prevention initiatives should broaden their scope to explore how to improve the delivery of the existing programs and what are the key contextual factors influencing the implementation process. This would enable a more profound understanding of why interventions seem to fail in certain circumstances and flourish in others.

Also, there is a clear need for studies examining program sustainment and how it relates to program effectiveness and factors predicting high implementation fidelity in the long run. Such studies would need to focus on the connection between the implementation fidelity and the outcomes, given that the implementation often varies across years (Herlitz et al., 2020; McIntosh et al., 2016a, b; Sainio et al., 2020). For example, the framework of sustainable educational change (Hubers, 2020) suggests that substantial changes in the everyday practices of a school promote individual and organizational changes over time. This process eventually also leads to improved student outcomes. Thus, in order to investigate the connection between program implementation and student outcomes, various mechanisms of change would need to be taken into account. Disentangling such effects can enhance our understanding on how to improve the programs, while also informing us of the needs for organizational or programmatic changes that can lead to more effective bullying prevention. Overall, future research should not be too short-sighted focusing merely on the immediate program outcomes, but also examine sustainability and investigate which aspects or components of the program are effective, and whether these components are feasible for the schools to implement in the long run.
